# The integration of spear and shield: a panoramic analysis of the blood circulation-promoting and hemostatic effects of *Panax notoginseng*

**DOI:** 10.1186/s13020-025-01100-6

**Published:** 2025-05-29

**Authors:** Xinyue Zhang, Chengxian Li, Guoyun Wang, Opoku Bonsu Francis, Hongda Wang, Aomei Sun, Han Wu, Xintong Yang, Pengzhi Dong, Wenke Zheng, Qilong Wang, Junhua Zhang

**Affiliations:** 1 Institute of Traditional Chinese Medicine, Tianjin University of Traditional Chinese Medicine, Tianjin, 301617 China; 2 National Key Laboratory of Chinese Medicine Modernization, Tianjin, China; 3 State Key Laboratory of Component-Based Chinese Medicine, Ministry of Education, Tianjin, China; 4 Haihe Laboratory of Modern Chinese Medicine, Tianjin, China; 5 Endocrinology Department, Fourth Teaching Hospital of Tianjin University of Traditional Chinese Medicine, Tianjin, China

**Keywords:** *Panax notoginseng*, Cardiovascular diseases, Circulate blood, Stop bleeding

## Abstract

*Panax notoginseng* is a widely used herb in traditional Chinese medicine renowned for its unique dual functionality in promoting blood circulation and stopping bleeding. However, the specific active components and the molecular mechanisms underlying the seemingly contradictory effects of *P. notoginseng* remain unclear. This article reviews the literature on *P. notoginseng* focusing on its use in conditions including cardiovascular diseases, promoting blood circulation, and hemostasis. It provides a panoramic view of the regulatory roles of the different active components of *P. notoginseng* in maintaining vascular homeostasis, inflammatory responses, and coagulation-fibrinolysis during blood-activating/hemostatic conditions. By summarizing the pharmacological effects of *P. notoginseng* as reported by other studies, we have conducted a preliminary exploration of the medicinal basis and mechanisms that underlie its seemingly contradictory blood-activating and hemostatic effects. This research lays the groundwork for future studies into exploring the underlying mechanisms of *P. notoginseng* in "stopping bleeding without causing stasis and promoting blood circulation without harming the blood," and its application in preventing and treating cardiovascular diseases.

**Graphical abstract:**

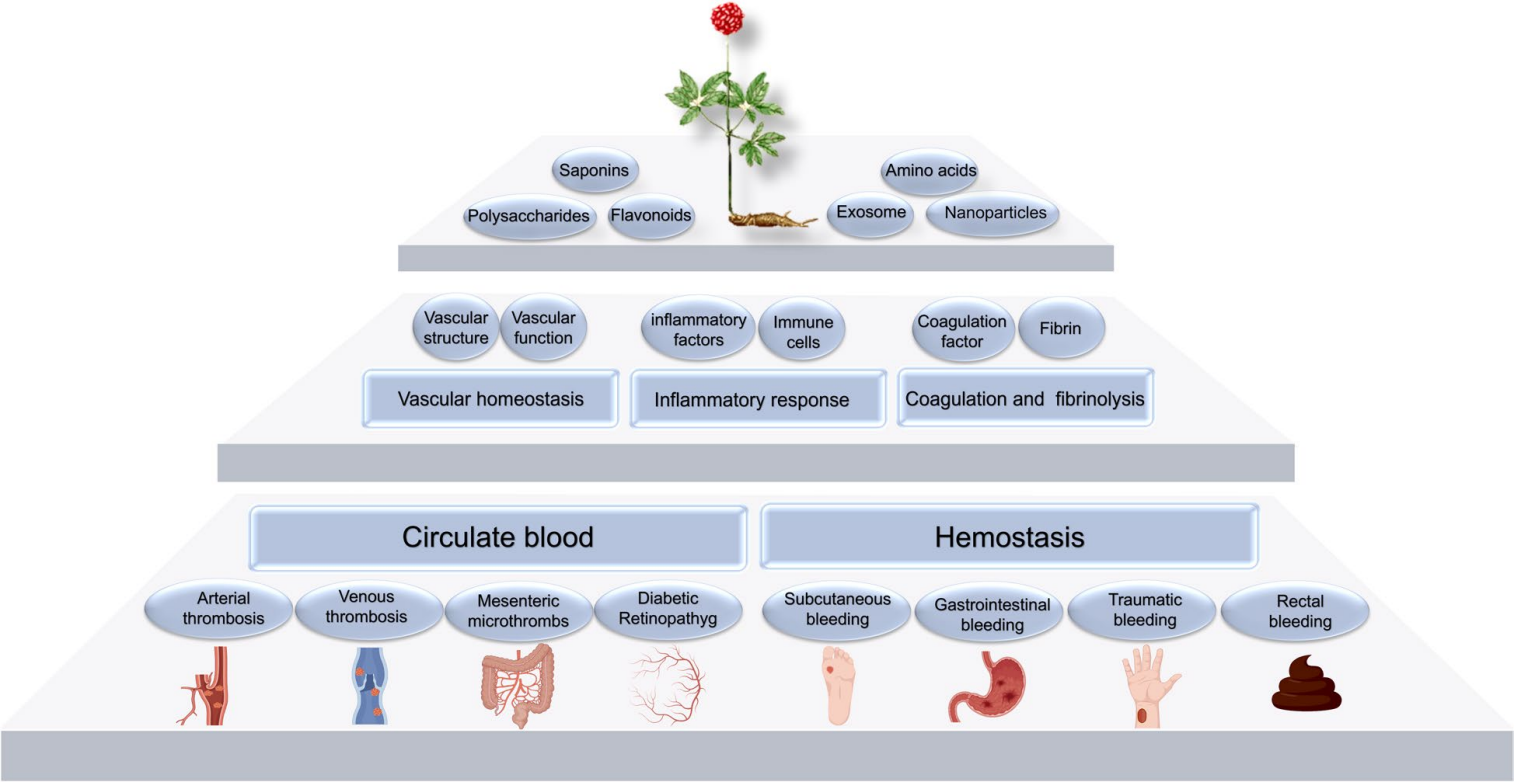

## Introduction

*P. notoginseng* (Sanqi) is the root and rhizome of *Panax notoginseng* (Burk.) F. H. Chen. In traditional Chinese medicine, *P. notoginseng* is recognized for its diverse therapeutic properties including improving blood circulation, stopping bleeding, reducing swelling, and alleviating pain. Phytochemical analysis of *P. notoginseng* has led to the identification of many constituents, including saponins, amino acids, flavonoids, essential oils, and carbohydrates [1, 2]. *P. notoginseng* helps maintain vascular homeostasis by improving several biological processes, including restoring the barrier function of endothelial cells [3], regulating vasodilation and vasoconstriction [4], and improving lipid metabolism [5]. It also promotes cell proliferation and migration, extracellular matrix remodeling, and vascular morphology [6], collectively preserving the normal structure and function of blood vessels. It has also been shown to play a significant role in modulating the inflammatory microenvironment by inhibiting the expression of inflammatory factors and reducing secondary damage caused by inflammation [7]. In another study, it was shown to suppress the release of cytokines from platelets, effectively reducing leukocyte aggregation [8]. In coagulation and fibrinolysis, *P. notoginseng* has been reported to regulate platelet function and coagulation factor activity [9]. It also promotes rapid clotting at sites of tissue damage while facilitating the timely dissolution of fibrin, preventing thrombosis, and effectively controlling bleeding [10].

The complex and diverse pharmacological activities of *P. notoginseng* make it a vital natural resource for treating various circulatory disorders and vascular diseases. This includes alleviating various forms of thrombosis, as well as stopping bleeding. Among the many traditional Chinese medicines that regulate blood flow, *P. notoginseng* stands out for its unique dual action of both promoting blood circulation and stopping bleeding. In Chinese medicine theory, this remarkable effect is often described as "stop bleeding without causing stagnation and promote circulation without harming the blood".

Thrombosis can lead to severe health issues, with deep vein thrombosis (DVT) being one of the most critical [11]. Currently approved anticoagulants for treating DVT work either by inhibiting factor Xa (FXa) to indirectly reduce thrombin production [12] (e.g., apixaban or rivaroxaban) or directly inhibiting thrombin activity (e.g., dabigatran), thereby blocking the coagulation cascade and inhibiting clot formation. While these treatments are effective in preventing clot formation and recurrence, they also increase the risk of bleeding complications [13, 14]. Similarly, in treating bleeding disorders such as hemophilia or other coagulation disorders, the excessive or indiscriminate use of clotting factor concentrates (Factor VIII or IX) can result in excessive coagulation and an increased risk of thrombosis [15]. It has also been shown that using antifibrinolytic drugs (such as tranexamic acid) or fibrinogen-rich hot gel to stop bleeding following acute or traumatic hemorrhage may cause thrombosis due to their strong hemostatic effects [16, 17]. In light of these challenges arising from current therapies, it is crucial to find drug alternatives that balance both pro-coagulant and anticoagulant effects, establishing a stable homeostasis that addresses the underlying condition without causing excessive clotting or bleeding. *P. notoginseng* offers a promising solution in this regard.

**Fig. 1:**
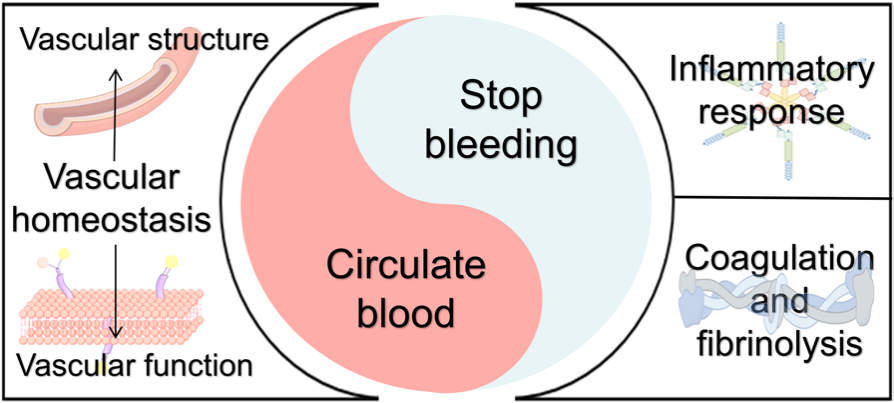
A comprehensive analysis of the blood-circulation and hemostatic effects of *P. notoginseng*. The central Taiji diagram symbolizes *P. notoginseng*'s dual ability to invigorate blood and stop bleeding, while the surrounding elements illustrate different perspectives of this comprehensive analysis, including vascular homeostasis, inflammatory responses, and coagulation-fibrinolysis processes

The medicinal parts of *P. notoginseng* are rich in bioactive compounds, such as notoginsenosides and flavonoids, which are effective in promoting blood circulation, stopping bleeding, and reducing inflammation. *P. notoginseng* is widely used for treating cardiovascular and cerebrovascular diseases due to its efficacy in managing thrombosis and hemorrhagic disorders [18]. Commonly used *P. notoginseng-*based formulations include Xueshuantong Injection, Xueshuantong Soft Capsules, *P. notoginseng* Tongshu Capsules, and Lulutong Injection [19–23]. Extensive studies on these formulations have shown that they are safe and effective alternative therapies that can improve the prognosis of patients with ischemic stroke, deep vein thrombosis, diabetic kidney disease, chronic renal insufficiency, etc. [24]. However, as a multi-component herbal medicine, *P. notoginseng*-based formulations also present several challenges in clearly defining their pharmacological mechanisms. Although extensive research has been conducted to uncover the molecular mechanisms of *P. notoginseng*, evidence linking its dual effects of promoting blood circulation and stopping bleeding and its active constituents remains limited. By examining three key aspects—vascular homeostasis, inflammation, and the coagulation-fibrinolysis processes—this review provides a comprehensive overview (Fig. 1) of the active components and mechanisms through which *P. notoginseng* exerts its dual effects. The goal is to uncover the pharmacological basis behind *P. notoginseng*’s ability to promote both circulation and hemostasis and to explore the mechanisms by which it balances these seemingly contradictory effects to achieve a therapeutic equilibrium.

## Panoramic analysis of *Panax notoginseng* in blood circulation

*P. notoginseng* can promote blood circulation, mainly reflected in promoting blood rheology and reducing blood clots [25], which are closely related to the blood vessels themselves. It is mainly affected by vascular homeostasis, inflammatory responses, and coagulation-fibrinolysis processes.

### Vascular homeostasis

The different components in *P. notoginseng* can act on several biological processes to achieve vascular homeostasis such as improving blood flow velocity, repairing vascular lesions, and other related processes. Vascular homeostasis refers to the maintenance of normal vascular structure and function. This homeostasis is essential for promoting healthy blood flow dynamics which ultimately reduces the risk of thrombosis.

#### Vascular structural regulation

**Fig. 2:**
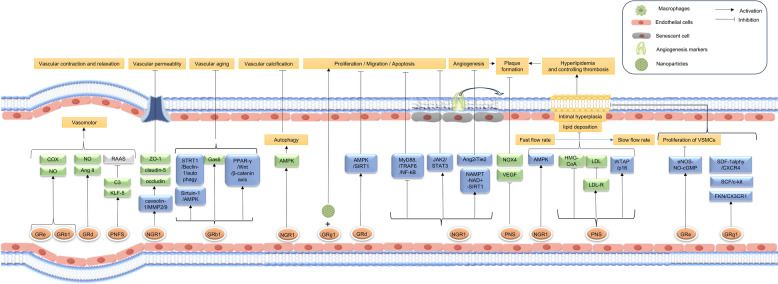
The regulatory effect of Sanqi on vascular homeostasis on the action of promoting blood circulation

The vascular wall is structurally organized into three concentric layers from the lumen outward: the intima, media, and adventitia [26]. The intima comprises a monolayer of endothelial cells (ECs) anchored to the basement membrane, with ECs lining the luminal surface and playing a pivotal role in maintaining vascular homeostasis. Vascular structural regulation, commonly termed vascular remodeling, encompasses three core biological processes: (1) Cell dynamics regulation, involving the precisely orchestrated balance of endothelial and smooth muscle cell proliferation, migration, and apoptosis to sustain vascular integrity; (2) Angiogenesis and extracellular matrix (ECM) remodeling, driven by dynamic interactions between cellular signaling and microenvironmental cues; and (3) Pathological structural alterations under disease conditions, characterized by degenerative changes such as ectopic calcification, cellular senescence, and neointimal hyperplasia. These maladaptive processes collectively impair vascular compliance and functionality, ultimately disrupting hemodynamics and exacerbating vascular pathologies. Multiscale regulatory mechanisms governing these processes critically influence circulatory efficiency and disease progression in the vasculature. The components in *P. notoginseng*, including *Panax notoginseng* saponins (PNS), Notoginsenoside R1 (NGR1), Ginsenoside Rg1 (Rg1), Ginsenoside Rb1 (Rb1), Ginsenoside Rd (Re) and Ginsenoside Re (Rd), all play critical roles in regulating vascular remodeling, and promoting blood circulation (Fig. 2).

##### *Panax notoginseng* saponins

Vascular endothelial growth factor (VEGF) is a key angiogenic growth factor that accelerates thrombus formation. NADPH oxidase 4 (NOX4) regulates the expression of VEGF and plays a critical role in vascular cell proliferation and migration. PNS can alleviate plaque angiogenesis and reduce atherosclerosis by decreasing the expression of VEGF and NOX4 [27]. The proliferation and migration of vascular smooth muscle cells (VSMC) can lead to intimal hyperplasia, resulting in increased vascular thickness, arterial stenosis, and increased risk of thrombosis. PNS has also been shown to inhibit intimal hyperplasia by regulating the WTAP/p16 signaling pathway through m6A modification [28]. PNS and its major components, GRg1 and NGR1, significantly enhance endothelial cell migration and angiogenesis in response to myocardial infarction (MI) injury [6].

##### Notoginsenoside R1

NGR1 regulates various physiological processes involved in vascular remodeling through multiple cellular signaling pathways, from cell proliferation to vascular system development. NGR1 activates the NAMPT-NAD-SIRT1 cascade, where SIRT1 inhibits DLL4-Notch signaling by deacetylating NICD and upregulating the expression of vascular endothelial growth factor receptor-2 (VEGFR-2). These processes collectively enhance the migration and proliferation of human brain microvascular endothelial cells (HBMECs), restore cerebral blood flow, and promote angiogenesis [29]. NGR1 promotes angiogenesis by activating the Ang-2/Tie-2 signaling pathway and enhancing the proliferation, migration, and tube formation of human umbilical vein endothelial cells (HUVECs), thereby alleviating ischemic symptoms [30]. NGR1 upregulates miR-147a to inhibit the MyD88/TRAF6/NF-κB signaling pathway, thereby suppressing endothelial cell proliferation, apoptosis, and injury, while simultaneously enhancing angiogenic capacity [31]. Myocardial infarction often leads to adverse cardiac remodeling and apoptosis of cardiac cells during chronic progression [32]. NGR1 has been shown in both in vivo and in vitro studies to reduce apoptosis and reduce the infarct size by activating the JAK2/STAT3 signaling pathway [33]. Endothelial senescence typically impairs endothelial-dependent dilation, angiogenesis, and barrier function, contributing negatively to thrombus formation [34]. NGR1 by regulating autophagy via the AMPK pathway, inhibits cellular senescence and slows down thrombosis formation, suggesting its potential as a therapeutic agent to mitigate endothelial senescence and maintain vascular homeostasis [35].

##### Ginsenoside Rg1

Vascular injury is often accompanied by intimal hyperplasia, which can reduce the diameter of the vascular lumen and restrict blood flow, impede vascular function, and increase the risk of thrombosis. Studies indicate that GRg1 prevents intimal hyperplasia in balloon-injured rats by modulating the SDF-1alpha/CXCR4, SCF/c-kit, and FKN/CX3CR1 pathways, thus maintaining vascular structure and homeostasis [36]. GRg1 nanoparticles targeting the transferrin receptor facilitate sustained release of GRg1, promoting migration and tubular formation of cerebral vascular endothelial cells, while also reducing the area affected by cerebral infarction [37]. In another study where a transferrin receptor-targeted peptide conjugated with a nano-carrier encapsulating hydrophobic GRg1 was reported to promote microvascular regeneration in the infarcted area [38].

##### Ginsenoside Rd

Diabetic retinopathy (DR), a quintessential microangiopathy of diabetes mellitus, is characterized by progressive vascular injury. Experimental studies demonstrate that GRd exerts cytoprotective effects through activation of the AMPK/SIRT1 signaling axis in hyperglycemic endothelial cells (EC), thereby attenuating apoptotic cascades and ameliorating DR and promoting microcirculation [39].

##### Ginsenoside Re

Studies on GRe regarding vascular homeostasis are limited, characterized by similarities and unique mechanisms compared to other saponins. Gao et al. found that ginsenoside GRe activates the eNOS-NO-cGMP signaling pathway, inhibits the proliferation of VSMCs, and prevents intimal thickening and progression of vascular lesions [40].

##### Ginsenoside Rb1

GRb1 primarily regulates vascular homeostasis by protecting endothelial cells and preventing vascular calcification. GRb1 reduces H₂O₂-induced endothelial dysfunction and inhibits vascular aging by stimulating the sirtuin-1/AMP-activated protein kinase (AMPK) pathway [41].

#### Vascular functional regulation

Vascular function is a complex physiological process involving the coordinated interactions of multiple cell types, molecular mechanisms, and signaling pathways to ensure the proper functioning of the circulatory system. Under normal conditions, vascular functions include endothelial barrier function [42], vasodilation and vasoconstriction regulation [43], and metabolic activities [44]. Endothelial dysfunction can lead to pathological conditions such as thrombosis, atherosclerosis, and inflammatory states, highlighting the critical importance of modulating vascular function in the treatment of cardiovascular diseases [45]. Recent pharmacological studies have demonstrated that PNS, along with NGR1, GRb1, GRg1, GRd, and GRe, all play a pivotal role in regulating vascular function (Fig. 2).

##### *Panax notoginseng* saponins

Studies have shown that oral administration of PNS can reverse hyperglycemia-induced endothelial dysfunction in mice aortic rings, primarily through the activation of AMPK and eNOS. Thus, increased eNOS activity promotes nitric oxide (NO) production. Nitric oxide is a known potent vasodilator that relaxes vascular smooth muscle, reducing vascular resistance and increasing blood flow, thereby improving circulation [46]. The two main PNS, GRe and GRb1 have been shown to induce vasodilation through NO and cyclooxygenase (COX) pathways [4]. Notoginseng flower saponins (PNFS) downregulate C3 and KLF-5 expression, inhibit the renin–angiotensin–aldosterone system (RAAS), improve endothelial function, and reduce blood pressure [47].

PNS also helps maintain vascular homeostasis by improving lipid metabolism, reducing low-density lipoprotein (LDL) and total cholesterol in the blood, thus minimizing lipid-associated endothelial damage. Lipid accumulation is a major contributing factor in the development of atherosclerosis (AS) and endothelial dysfunction. PNS prevents lipid deposition on the vascular walls by inhibiting key enzymes in hepatic cholesterol biosynthesis, such as 3-hydroxy-3-methylglutaryl coenzyme A reductase (HMG-CoA reductase). This leads to a reduction in total cholesterol levels in the blood. PNS also regulates lipoproteins by increasing the expression of LDL receptors, which promotes the clearance of LDL-C from the blood, while simultaneously raising high-density lipoprotein (HDL) levels. HDL helps transport cholesterol from the vessel walls back to the liver for metabolism and excretion, thereby reducing cholesterol levels and preventing endothelial damage.

##### Notoginsenoside R1

The vascular endothelium has selective permeability allowing it to mediate the transport of specific substances across blood vessels. The permeability of the endothelial barrier is intricately related to the regulation of vasodilation and vasoconstriction, all of which together influence hemodynamics and tissue homeostasis. Increased endothelial permeability can alter hemodynamics and elevate the risk of thrombosis. Studies have shown that NGR1 reduces OGD-induced endothelial barrier permeability, restores the expression of ZO-1 and claudin-5 on the cell membrane and cytoplasm, and mediates the redistribution of occludin and caveolin-1 on the membrane in actin cytoskeleton regions. By modulating the caveolin-1/MMP2/9 pathway, NGR1 regulates the degradation and redistribution of ZO-1, claudin-5, and occludin, thus controlling endothelial permeability and signaling. This helps maintain vascular homeostasis and reduces thrombus volume in cerebral infarction [48].

Other studies have indicated that NGR1 can reduce endothelial damage by regulating lipid metabolism. It activates phosphorylated AMPK, promotes fatty acid oxidation, and inhibits lipid synthesis, thereby reducing intracellular lipid accumulation and maintaining vascular homeostasis [5]. NGR1 regulates brain small-molecule metabolism and enhances cerebral perfusion, thereby effectively ameliorating focal cerebral ischemia [49].

##### Ginsenoside Rb1

GRb1 primarily regulates vascular homeostasis by protecting endothelial cells and preventing vascular calcification. GRb1 reduces H₂O₂-induced endothelial dysfunction and inhibits vascular aging by stimulating the sirtuin-1/AMP-activated protein kinase (AMPK) pathway [41].

Additionally, GRb1 alleviates ox-LDL-induced endothelial aging through the SIRT1/Beclin-1/autophagy axis [50]. GRb1 has also been shown to mitigate age-related vascular damage by regulating the Gas6 pathway and improves chronic kidney disease-related vascular calcification via the PPAR-γ/Wnt/β-catenin axis [51, 52]. In another study, GRb1 was shown to inhibit vascular smooth muscle cell calcification through androgen receptor-mediated Gas6 transactivation and its antagonistic effect in prostate cancer cells [53].

##### Ginsenoside Rg1

In the aorta of mice exposed to chronic intermittent hypoxia, vasodilation and vasoconstriction were seen to be abolished and both of these effects were restored by GRg1 treatment [54]. Additionally, GRg1 has been shown to help maintain vascular homeostasis in diabetic states. By reducing the increased expression of heparanase, GRg1 alleviates high glucose(HG)-induced endothelial barrier dysfunction [55].

##### Ginsenoside Rd

GRd exerts beneficial effects by maintaining physiological NO signaling pathways, augmenting angiotensin II production, suppressing pathological vascular constriction, and protecting aortic endothelial cells [56].

##### Ginsenoside Re

GRe increases endothelial Ca^2+^-activated K^+^ outward currents, which stimulates NO production and promotes vasodilation.

### Inflammation and oxidative stress

**Fig. 3:**
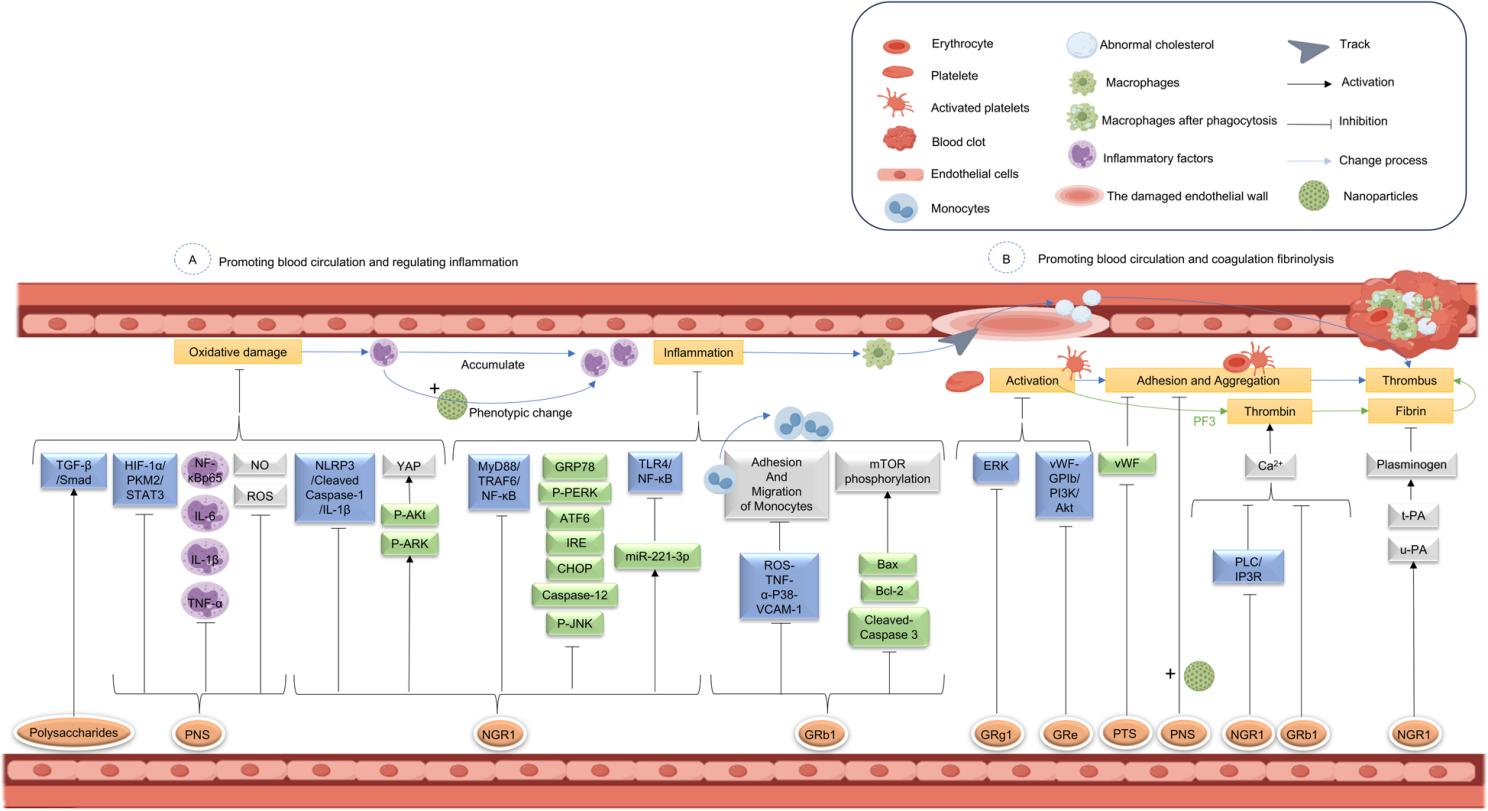
The effect of Sanqi on the regulation of inflammation, coagulation and fibrinolysis on the action of promoting blood circulation

Inflammation is a defensive response of the body to external stimuli such as injury, pathogens, or irritants, aimed at eliminating harmful agents and promoting tissue repair. Despite its beneficial effects, the interactions between platelets, neutrophils, and the formation of neutrophil extracellular traps (NETs), monocytes, and macrophages under pathological conditions have been heavily implicated in the occurrence of thrombo-inflammation [57]. The inflammation occurring during thrombosis primarily manifests as the activation of platelets and innate immune cells, creating a cycle that further activates the complement system and coagulation cascade [58]. Additionally, the development of cardiovascular diseases, such as atherosclerosis, is often associated with endothelial dysfunction which mainly arise from abnormal inflammatory responses and oxidative stress. Therefore, inflammation and oxidative stress are key contributing factors in thrombosis, interacting through the secretion of pro-inflammatory cytokines, as well as the adhesion and migration of monocytes. For instance, saponins (such as PNS, GRb1, and NGR1), polysaccharides, exosome and related nanoparticles in *Panax notoginseng*, along with quercetin (QUE), have been shown to exhibit significant anti-inflammatory and blood-regulating effects that may help modulate these pathological processes (Fig. 3).

#### *Panax notoginseng* saponins

The secretion of pro-inflammatory cytokines enhances the rate of clot formation, promoting thrombosis [59]. For example, PNS effectively inhibit the NF-κB signaling pathway, thereby reducing the expression of pro-inflammatory factors such as NF-κB p65, IL-6, IL-1β, TNF-α, and Calpain1 proteins in the aortic root tissues of apoE−/− mice, which decreases lipid deposition and the formation of atherosclerotic plaques [7]. IL-1β is a key inflammatory cytokine that promotes atherosclerosis by facilitating macrophage migration and increasing the risk of clot formation. The use of PNS can effectively reduce the expression of IL-1β, thereby decreasing the formation of thrombus [60]. Additionally, PNS exerts anti-inflammatory effects and improves tissue blood supply by lowering levels of NO and ROS while enhancing the activity of immune cells [61]. In neurological disease models, PNS has been shown to alleviate inflammation caused by activated microglia by inhibiting the HIF-1α/PKM2/STAT3 signaling pathway, thus improving local cerebral blood flow.

#### Notoginsenoside R1

NGR1 regulates redox status by activating PARP, thereby reducing oxidative damage and apoptosis [62]. Also, NGR1 effectively protects endothelial cells by blocking the activation of the MyD88/TRAF6/NF-κB signaling pathway under high-glucose states, thereby inhibiting oxidative stress and inflammatory responses [31]. NGR1 can also inhibit the NLRP3/Cleaved Caspase-1/IL-1β inflammatory pathway, effectively reducing endothelial cell damage by suppressing inflammation [63]. Furthermore, NGR1 increases the expression of miR-221-3p in ox-LDL-induced HUVEC, thus inhibiting the activation of the TLR4/NF-κB pathway. These process modifications ultimately reduce apoptosis, inflammation, and oxidative stress while exerting anti-atherosclerotic and anti-thrombotic effects [64]. Under ischemic conditions, NGR1-based nanoparticles have been shown to increase the levels of p-Akt and pERK, thus promoting the nuclear translocation of YAP and resulting in reduced infarct areas [65]. Ischemia–reperfusion injury (I/R) refers to the damage caused by excessive free radicals attacking cells in tissues that regain blood supply after vascular obstruction, such as from a thrombus. NGR1 decreases the protein expression of endoplasmic reticulum stress (ERS) response proteins GRP78, P-PERK, ATF6, and IRE while inhibiting the expression of pro-apoptotic proteins CHOP, Caspase-12, and P-JNK to prevent apoptosis and delay the onset of ERS.

#### Ginsenoside Rb1

Monocytes transform into foam cells after ingesting lipids in the arterial wall. This process not only promotes inflammation but is also a critical step in the development of atherosclerosis. GRb1 reduces the adhesion and migration of monocytes by inhibiting the ROS-TNF-α-p38-VCAM-1 signaling pathway, thereby suppressing inflammation, protecting endothelial cells, and alleviating lipid retention and ultimately, thrombus formation [66]. Additionally, Caspase-3 serves as a crucial signaling protein in the death receptor pathway, playing an important role in the regulation of apoptosis and inflammation. In a myocardial ischemia–reperfusion (I/R) injury model, GRb1 downregulated the expression of Bax, Bcl-2, and cleaved-caspase 3 while activating mTOR phosphorylation, effectively inhibiting apoptosis and the inflammatory response. This mechanism helps mitigate cellular and tissue damage that occurs when blood flow is restored following blockades caused by thrombosis or other obstructive mechanisms [67].

#### *Panax notoginseng* polysaccharides

After extraction, the residues of *P. notoginseng* contain rich polysaccharides. Recent studies have shown that the homogeneous polysaccharide PNP-20, isolated using a graded precipitation method, can alleviate intestinal damage and inflammatory cell infiltration in LPS-induced mouse colitis [68]. Two new polysaccharides, MRP5 and MRP5A, isolated from the roots of *P. notoginseng* have demonstrated significant antioxidant properties and lifespan extension in *Caenorhabditis elegans* [69]. Fermented *P. notoginseng* polysaccharides can activate the TGF-β/Smad signaling pathway to inhibit collagen and elastin damage induced by H₂O₂, thereby protecting the skin tissues from oxidative damage [70]. Additionally, *Panax notoginseng* polysaccharides can protect myocardial cells from hypoxia-induced damage by regulating mitochondrial function and also reduce ischemia–reperfusion (IR) injury [71, 72].

#### Quercetin from *Panax notoginseng*

QUE reduces oxidative damage and inflammatory responses to protect the great saphenous vein (GSV) and inhibits abnormal thickening of the venous endothelium by suppressing cell proliferation [73].

#### Nanoparticles of *Panax notoginseng*

*Panax notoginseng-*derived exosome-like nanoparticles could reduce the area of cerebral infarction by altering the inflammatory phenotype of microglia [74].

### Coagulation and fibrinolysis

Blood coagulation is a complex yet sequential chain of events in which clotting factors are continuously activated, forming a systematic and highly regulated pathway that ultimately leads to the formation of a blood clot to prevent excessive bleeding. The coagulation process is divided into intrinsic and extrinsic pathways. The extrinsic pathway is related to tissue factor (TF) exposed outside the vessel due to vascular injury, while the intrinsic pathway is a positive feedback system that involves factors within the blood vessels, amplifying the coagulation process. These two pathways converge to produce thrombin, which generates fibrin. Thrombin cleaves soluble fibrinogen to form insoluble fibrin, further activating platelets and initiating the propagation of the clot, ultimately resulting in thrombus formation.

Calcium ions serve as a crucial link for the localization and interaction of clotting factors. In the liver, vitamin K facilitates the gamma-carboxylation of clotting factors containing glutamic acid, rendering them negatively charged and allowing them to bind with positively charged calcium ions. Simultaneously, calcium ions connect to the negatively charged surface of cell membranes, initiating the precise coagulation process at the site of vascular injury. Thus, regulating calcium ion levels may prevent or mitigate myocardial ischemia–reperfusion injury [75].

*P. notoginseng* regulates both the intrinsic and extrinsic pathways during the coagulation process. The primary mechanisms involve regulating the interactions among thrombin, fibrin, platelets, and calcium ions. Various components in *P. notoginseng*, including PNS, NGR1, GRb1, GRe, GRg1, Ginsenoside Rg3 (GRg3), *Panax notoginseng* triol saponins (PTS), and QUE, exert their effects on blood circulation through the coagulation and fibrinolytic systems (Fig. 3).

#### *Panax notoginseng* saponins

PNS modulates the expression of COX-2, leading to an increase in the concentration of 6-keto-PGF1α in HUVECs. Simultaneously, it downregulates COX-1 expression in platelets and decreases the concentration of TXB2, thereby inhibiting platelet activation and adhesion and ultimately reducing the risk of thrombus formation [76]. Novel Breviscapine nanocrystals modified with PNS exhibit enhanced antiplatelet aggregation activity [77].

#### Notoginsenoside R1

NGR1 has been shown to enhance fibrinolysis by stimulating tissue-type plasminogen activator (t-PA) and urokinase-type plasminogen activator (u-PA), resulting in an increased production of plasminogen, decreased fibrinogen deposition and lowering the risk of thrombus formation [9]. Additionally, NGR1 inhibits the PLC/IP3R pathway and the release of Ca^2^⁺ from the endoplasmic reticulum (ER), alleviating hypoxic-ischemic encephalopathy [78].

#### Ginsenoside Rb1

GRb1 reduces intracellular calcium levels, enhancing cell viability and decreasing the ischemic area of myocardial infarction [79]. GRb1 exerted anti-platelet and anti-thrombotic effects at high shear rates via Piezo1 channels [80].

#### Ginsenoside Rg1

GRg1 inhibits platelet activation by suppressing the ERK pathway, which reduces coagulation factors, inhibits thrombin production, and decreases fibrinogen deposition, ultimately attenuating arterial thrombus formation in vivo [81].

#### Ginsenoside Re

GRe prevents thrombus formation by inhibiting platelet activation through the vWF-GPIb/PI3K/Akt pathway, without increasing the risk of bleeding [10].

#### Ginsenoside Rg3

GRg3 inhibits the influx of Ca^2+^ induced by thapsigargin and reduces the increased phosphorylation of ERK2 caused by thrombin, thereby suppressing platelet aggregation [82].

#### *Panax notoginseng* triol saponins

PTS can reduce the adhesion of platelets to damaged vascular endothelium mediated by vWF, thereby exhibiting anti-platelet aggregation and anti-thrombotic effects [8].

#### Quercetin from *Panax notoginseng*

Network pharmacology analysis indicated that quercetin in *Panax notoginseng* exerts its anti-deep vein thrombosis effects through the RB1 and TP53 pathways [83]. GRb1 suppresses platelet aggregation and regulates the production of plasminogen activators, thereby promoting thrombus dissolution. In endothelial cells, Tp53 regulates the anticoagulant function of vascular endothelial cells by upregulating coagulation inhibitors (such as tissue factor pathway inhibitors and endothelial-dependent anticoagulants), thereby reducing blood clotting. Tp53 also enhances fibrinolysis by activating tissue plasminogen activator (tPA) and its receptor, preventing the formation of deep vein thrombosis.

## Panoramic analysis of *Panax notoginseng* in hemostasis

**Fig. 4:**
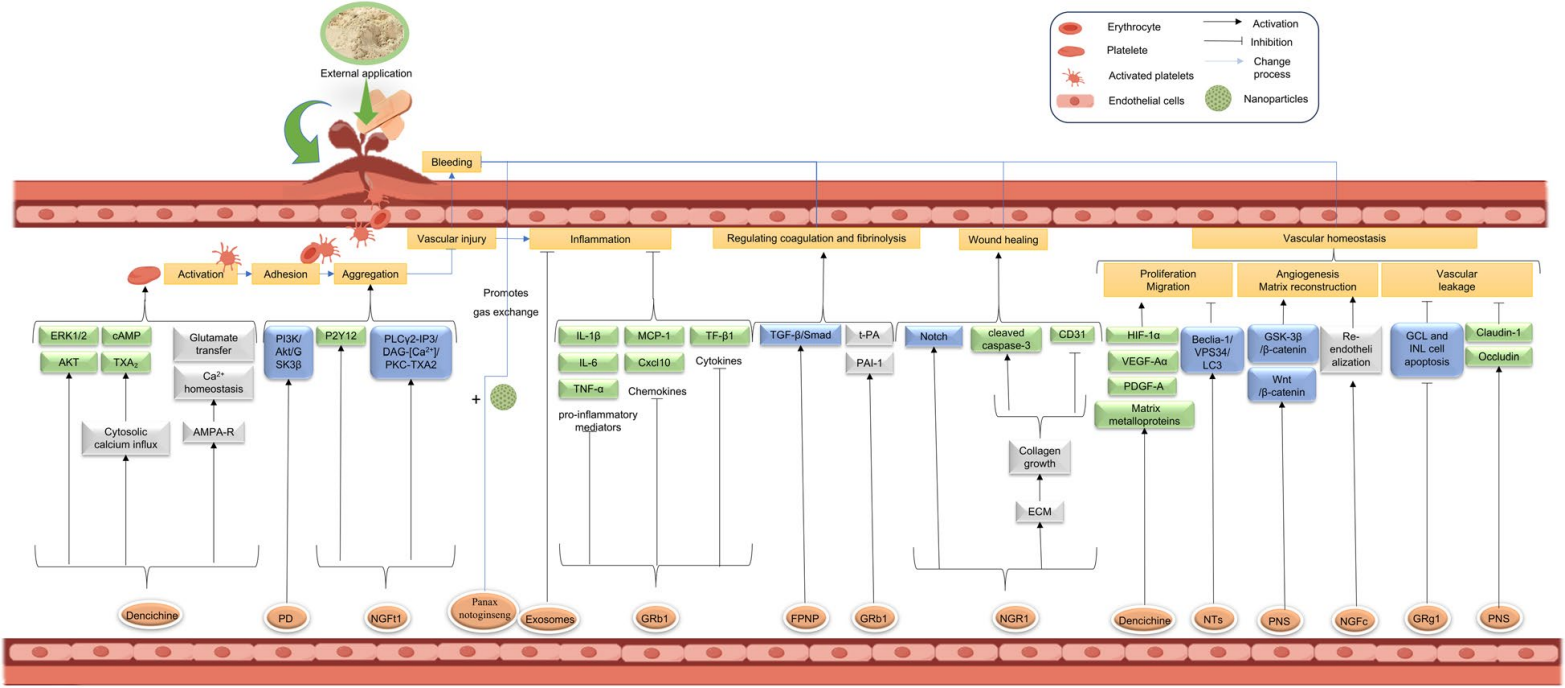
Hemostatic mechanisms of *P. notoginseng*

Hemostasis refers to the process of repairing bleeding at the site of vascular injury, primarily achieved by forming a blood clot that seals the damaged area. This process involves vasoconstriction which narrows the lumen of the blood vessels to reduce blood loss caused by vascular damage, thus facilitating vascular repair. After the vascular injury, platelets arrive at the injured site and adhere to exposed collagen and other extracellular matrix components. Once adhered, platelets activate and release various chemical signals that attract additional platelets to the site, forming a platelet plug. Concurrently, coagulation factors initiate a series of enzymatic reactions that ultimately convert fibrinogen in the plasma into fibrin. Fibrin then forms a mesh-like structure that stabilizes and reinforces the platelet plug, effectively stopping blood loss and initiating the tissue healing process. Components in *P. notoginseng* that have been shown to possess hemostatic properties include saponins such as PNS, NGR1, GRb1, Notoginsenoside Ft1 (NGFt1), and polysaccharides. These active constituents participate in the regulation of the coagulation process through various pathways related to vascular homeostasis, structural remodeling, inflammation and oxidative stress, and coagulation and fibrinolysis. Figure 4 illustrates the pathological processes following vascular injury and bleeding, as well as the therapeutic mechanisms of both oral and topical administration of *P. notoginseng* (Fig. 4).

### Vascular homeostasis

Hemostasis and vascular homeostasis are intrinsically interconnected. During hemostasis, the vasculature orchestrates injury repair and minimizes blood loss via structural remodeling and functional modulation. This equilibrium can be modulated by bioactive compounds derived from *Panax notoginseng*, including PNS, NGR1, GRg1, Notoginseng Triterpenes (NTs), Notoginsenoside Fc (NGFc), dencichine, and combined nanomaterials, which reinforce the coupling between hemostasis and vascular homeostasis by enhancing vascular compliance and facilitating tissue regeneration.

#### Vascular structural regulation

The cellular processes of proliferation, migration, and apoptosis can regulate vascular structural changes, promote angiogenesis and matrix remodeling, increase blood flow to the injured area, and play a crucial role in wound healing. These processes not only facilitate the repair of damaged tissues but also promote endothelialization following vascular injury, thereby accelerating vascular repair and wound healing. This structural regulation is essential for effectively closing wounds and repairing tissues and is crucial for preventing excessive bleeding and supporting the body’s natural clotting mechanisms. Understanding these aspects lays the foundation for exploring the potential mechanisms driving vascular repair and wound healing, and highlights the importance of vascular structural regulation in the study of hemorrhagic diseases. The components PNS, NGR1, NGFc, NTs, dencichine, and combined nanomaterials play a crucial role in promoting wound healing and tissue repair.

##### *Panax notoginseng* saponins

PNS can activate the Wnt/β-catenin signaling pathway to enhance endothelial progenitor cell (EPC) angiogenesis [84], resulting in neovascularization which provides increased blood flow to injured areas, enhancing the supply of oxygen and nutrients, while accelerating the reconstruction of damaged vascular walls. Additionally, PNS promotes endothelial cell proliferation, invasion, migration, and angiogenesis, inhibits cell apoptosis, facilitates wound healing, and enhances matrix remodeling in vivo [85].

##### Notoginsenoside R1

NGR1 can activate the Notch pathway involved in the healing process of diabetic ulcers. It has also been shown to enhance the secretion of ECM, promote collagen growth, increase the expression of CD31, and reduce the expression of cleaved caspase-3 to facilitate wound healing in diabetic complications [86].

##### Notoginsenoside Fc

Hyperglycemia in diabetic conditions plays a crucial role in compromising endothelial integrity, resulting in delayed reendothelialization and excessive neointimal formation. NGFc enhances vascular repair and promotes wound healing in diabetic rats by accelerating reendothelialization after vascular injury, mediated through autophagy activation [87].

##### Notoginseng triterpenes

Random flaps are widely used to repair wounds and improve shape and functional reconstruction. They are characterized by their independence from specific vascular axes [88]. While these structures offer some flexibility, the uncertainty in vascular distribution can lead to insufficient blood supply to distal tissues, increasing the risk of tissue necrosis [89]. NTs can activate the Beclin-1/VPS34/LC3 signaling pathway, promoting cell proliferation and migration, which effectively enhances blood perfusion and improves the survival area of flaps [90].

##### Dencichine

Dencichine, also known as L-β-oxalylaminoanaline (L-ODAP), can increase the expression of HIF-1α, VEGF-A, PDGF-A, and matrix metalloproteins, thereby stabilizing cell proliferation and migration to promote wound healing [91, 92].

##### *P. notoginseng* nanofiber membrane

By combining electrospinning technology with *P. notoginseng*, a nanofiber membrane was fabricated, which promotes gas exchange at the wound site, enhances fibroblast adhesion, improves vascularization, and accelerates wound healing [93].

#### Vascular functional regulation

The regulation of vascular function is crucial for maintaining tissue homeostasis and mitigating pathological progression in the context of hemostasis. These processes involve dynamic control over vascular permeability, thromboresistance, and barrier integrity, collectively preventing abnormal blood leakage and stabilizing the microcirculatory environment. By regulating endothelial cell interactions and balancing pro-hemostatic and anti-hemostatic factors, vascular function regulation can counteract pathological cascades to a certain extent. Targeting vascular function in hemostatic therapies has shown potential for restoring physiological balance in states of vascular injury, especially when structural deterioration follows functional impairment. PNS and GRg1 have been demonstrated to reduce vascular leakage and promote hemostasis.

##### *Panax notoginseng* saponins

PNS increases the thickness of the retinal inner nuclear layer by upregulating the expression of claudin-1 and occludin proteins. These proteins, when expressed, reduce the proliferation of acellular capillaries in the retina and alleviate damage to the blood-retinal barrier (BRB) contributing to maintaining retinal vascular homeostasis and potentially aiding in the treatment of retinal diseases such as diabetic retinopathy or retinal vascular leakage [94].

##### Ginsenoside Rg1

GRg1 regulates retinal permeability and structure by reducing the ganglion cell layer (GCL) and the inner nuclear layer (INL) cell apoptosis, decreasing vascular leakage, and inhibiting diabetes-induced retinal damage [95].

### Regulating cell apoptosis and inflammation

After vascular injury and bleeding, a series of pathological processes are initiated, one of which is the apoptosis of endothelial cells, leading to oxidative stress and inflammatory responses. The saponin components in *P. notoginseng*, such as GRb1, GRd, and fermented *Panax notoginseng* polysaccharides (FPNP), along with its derived exosomes, possess anti-inflammatory properties aiding in vascular repair and promoting hemostasis.

#### Ginsenoside Rb1

GRb1, the primary anti-inflammatory component of *P. notoginseng* can inhibit the gene expression of the pro-inflammatory mediators (IL-1β, IL-6, TNF-ɑ), chemokines (MCP-1, CXCL10), and cytokines (TGF-β1) and also inhibit the expression of the proteolytic enzyme MMP-9 maintaining strong anti-inflammatory activities [96]. GRb1 exerts a therapeutic effect on intracerebral hemorrhage by ameliorating hippocampal neuroinflammation via inactivating the TLR4/NF-kB pathway [97].

#### Ginsenoside Rd

The cGAS/STING pathway is a core signaling pathway of the innate immune system, closely related to inflammatory responses. GRd alleviates early brain injury by inhibiting ferroptosis through cGAS/STING/DHODH pathway after subarachnoid hemorrhage [98].

#### Fermented *Panax notoginseng* polysaccharides

FPNP can attenuate oxidative stress by inhibiting hydrogen peroxide (H₂O₂)-induced damage to collagen and elastin via activating the TGF-β/Smad signaling pathway [70].

#### *Panax notoginseng* serum exosomes

PNS serum exosomes can effectively improve the inflammation of brain tissue in rats with cerebral hemorrhage and reduce cerebral hemorrhage damage [99].

### Hemostasis, coagulation, and fibrinolysis

#### Regulation of coagulation and fibrinolysis

Platelets and clotting factors are the primary components that facilitate hemostasis. During vascular injury, blood flows into surrounding tissues, where exposed collagen binds to and activates platelets leading to platelet aggregation. Simultaneously, endothelial cells at the injured site are prompted to release additional vasoconstrictors to constrict blood vessels, reducing blood flow to limit blood loss. Hemostasis can thus be achieved by reducing NO levels in plasma or increasing histamine, which leads to vasoconstriction. Activated platelets not only serve as coagulation sites on their surfaces but also form platelet aggregates that adhere to and cluster with endothelial cells. These aggregates are reinforced by fibrin, the final product of the coagulation cascade. Subsequently, nearby platelets are recruited and activated, creating a positive feedback mechanism that accelerates the hemostatic process. Together, these processes minimize damage to the surrounding tissues that are most directly affected [100–102]. Components in *P. notoginseng* that have been shown to regulate coagulation and fibrinolysis include GRb1, NGFt1, 20(S)-panaxadiol (PD), and dencichine.

##### Ginsenoside Rb1

T-PA and plasminogen activator inhibitor-1 (PAI-1) are key regulators of the fibrinolytic system. GRb1 can block the effects of ox-LDL thereby increasing the levels of t-PA and PAI-1 and inhibiting fibrinolysis [103].

##### Notoginsenoside Ft1

NGFt1 is a dammarane triterpene glycoside isolated from *P. notoginseng*. It has been shown to potentiate the PLCγ2-IP3/DAG-[Ca^2+^]/PKC-TXA2 signaling pathways stimulated by thrombin, collagen, or thrombin, highlighting its usefulness in promoting clotting in clotting deficient conditions like hemophilia. It also shortened the bleeding time and normalized the prolonged bleeding time induced by aspirin administration [104]. NGFt1 can enhance platelet aggregation, induce the proliferation of cultured HUVECs, promote GRe epithelialization, and accelerate wound healing [105, 106].

##### Dencichine

Dencichine can reduce the calcification time and the in vitro clotting time [92, 107].

#### Platelet regulation

##### Dencichine

Dencichine regulates platelet production by promoting megakaryocyte adhesion, migration, and pro-platelet formation through the ERK1/2 and Akt signaling pathways, thus preventing blood loss while increasing platelet counts [108]. Dencichine modulates platelet intracellular calcium influx, cyclic adenosine monophosphate (cAMP) production, and TXA2 release, thus activating AMPA receptors on platelets to enhance hemostasis [109]. The components NGFt1 and PD found in *P. notoginseng* contribute to hemostasis by regulating calcium ions and platelets.

##### Notoginsenoside Ft1

NGFt1 can promote platelet aggregation, and studies suggest that its mechanism may involve the activation of the P2Y12 pathway [110].

##### 20(S)-panaxadiol (PD)

PD influences calcium signaling and activates the PI3K/Akt/GSK3β signaling pathway, inducing platelet aggregation and promoting hemostasis [111].

## Discussion

**Fig. 5:**
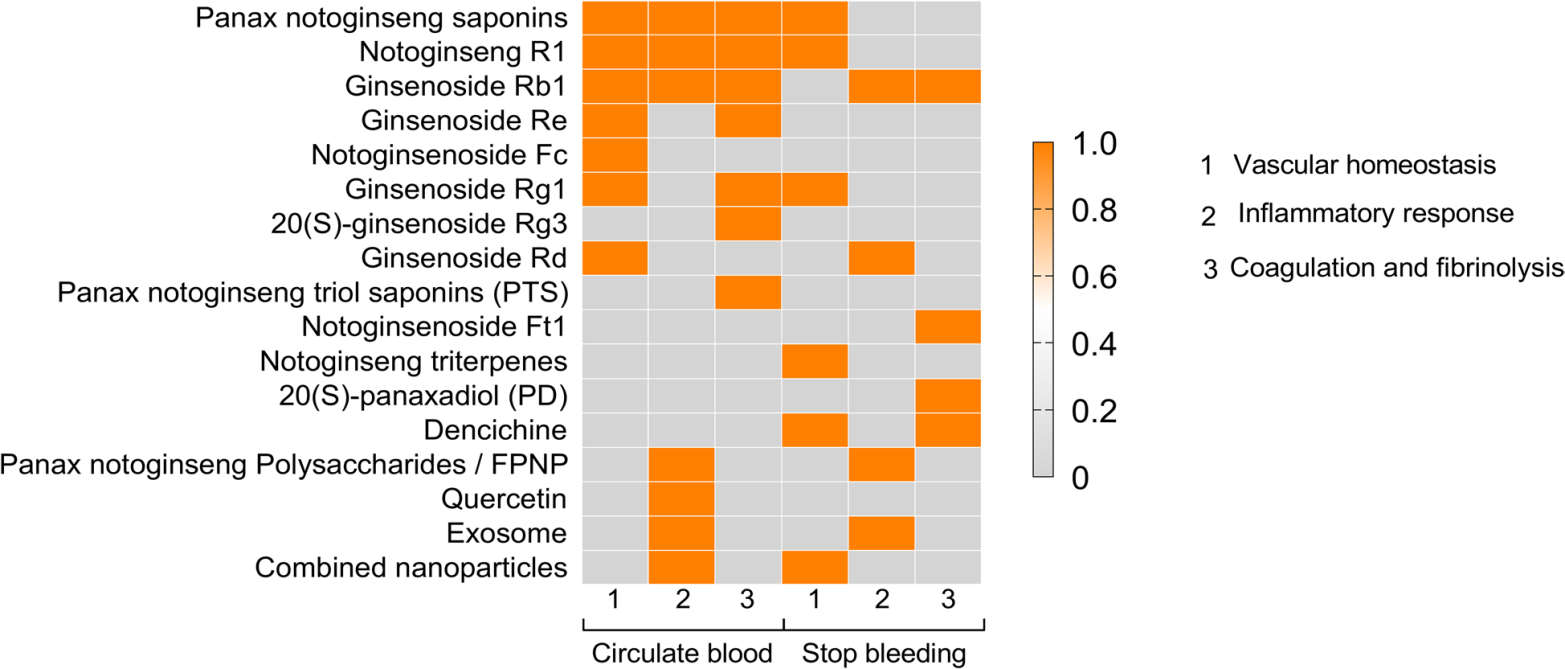
*Panax notoginseng* panoramic analysis heatmap. This heatmap illustrates the involvement of active components from *P. notoginseng* in promoting blood circulation and hemostasis. The numbers 1, 2, and 3 represent vascular homeostasis, inflammatory responses, and coagulation-fibrinolysis processes, respectively.Yellow indicates that the active component exerts its therapeutic effects through this pathway, while grey denotes the absence of reported studies to date, which does not imply the inherent inefficacy of the pathway

*P. notoginseng*, a highly valued traditional Chinese medicine, has been used for medical interventions for over four centuries. In China, the market valuation for *P. notoginseng* has exceeded 10 billion yuan [112]. It holds significant potential for widespread application in modern medicine and a vast medical market. In this article, we have reviewed the latest research on the representative bioactive compounds of *P. notoginseng* and their various pharmacological effects (Table 1). We found that most of the active compounds with hemostatic and blood-circulation effects were primarily saponins. Specifically, PNS, NGR1, GRg1, GRg3, PTS, GRb1, GRe, and GRd exhibit blood-circulation properties, while PNS, NGR1, GRg1, NGFc, GRb1, GRd, NGFt1 and PD demonstrate hemostatic effects (Fig. 5). In addition, nano drug delivery systems are of great significance for enhancing the efficacy of *Panax notoginseng*, especially in the treatment of cerebral infarction [65]. For example, nanoparticle carriers can increase the blood–brain barrier penetration efficiency of *Panax notoginseng*'s active ingredients [38]. The application of nanotechnology, such as nano-controlled release systems and nanocrystallization [37, 77], in the research of new drug delivery forms for *Panax notoginseng* can significantly improve drug solubility and therapeutic efficacy. Exosomes, as nanoscale vesicles released by cells, also possess the advantages of nano drug delivery systems [74]. The proteins, RNA molecules, and other components contained in the extracellular vesicles of *Panax notoginseng* have the potential to regulate immune responses, promote tissue repair [93, 99], and hold broad research prospects. The unique pharmacological feature of *P. notoginseng* is its dual ability to invigorate blood and promote hemostasis, allowing it to address both thrombosis and hemorrhagic conditions without causing excessive bleeding or clotting—an advantage not found in other medications [17]. Furthermore, *P. notoginseng* shows promising prospects in conditions where both blood circulation and coagulation systems are impaired, such as traumatic coagulopathy and disseminated intravascular coagulation.

**Table 1:** The pharmacological effect and mechanism of the main ingredients of *Panax notoginseng*

Component	Function		Experimental model	Dosage	Administration method	Pathological changes	Mechanism	References
*Panax notoginseng* saponins (PNS)	Circulate blood	Vascular homeostasis	Rat aorta rings	0.2, 0.4, 0.6, 0.8 mg/mL	Thoracic aortas	Reduced the tonic contraction (% of NE) at all doses investigated	Induces vasodilation through NO and cyclooxygenase (COX) pathways	[4]
			Spontaneously hypertensive rats (SHR)	30, 60, 120 mg/kg	oral daily gavage for six weeks (whole extract of PNFS	Reduce blood pressure, face temperature, and vertigo time, increase grip strength and improve dyslipidemia in rats with MH	Downregulates C3 and KLF-5 expression, inhibits the renin–angiotensin–aldosterone system (RAAS), improves endothelial function, and reduces blood pressure	[47]
			Vascular smooth muscle cells (VSMCs)	0.5, 1.0, 1.5, 2.0 μM	Cells incubated with TPNS at various concentrations for approximately 24 h	Repressed the viability, proliferation, and migration of hVSMCs	Inhibits intimal hyperplasia by regulating the WTAP/p16 signaling pathway through m6A modification	[28]
			Venous blood	0.375, 2.5, 5 mg/mL	blood samples of healthy subjects incubated with indicated concentrations of PNS for 10 min	Suppressed platelet aggregation	Enhances endothelial cell migration and angiogenesis in response to MI injury	[6]
		Inflammatory response	ApoE^−/−^ mice	60 mg/kg/d	mice were randomized to receive administration for 8 weeks	Inhibited plaque area, IMT, and lipid deposition in atherosclerotic lesions	Modulate fluid shear stress to mitigate the impact of thrombi on blood vessels	[60]
			ApoE^−/−^ mice	60, 180 mg/kg/d	Administered orally for 8 weeks	The arrangement of the aortic root tissues tended to be normal, while cell morphology was restored, and lipid depositions were reduced	Inhibits the NF-κB signaling pathway, thereby reducing the expression of pro-inflammatory factors such as NF-κB p65, IL-6, IL-1β, TNF-α, and Calpain1 proteins in the aortic root tissues of apoE^−/−^ mice	[7]
			A porcine lung cell line (3D4/2 cells)	100, 200, 400 μg/mL	The infected cells were grown in complete RPMI-1640 medium supplemented with PNS	Enhanced the activity of immune cells, exert anti-inflammatory effects and improved tissue blood supply	Reduce the levels of NO and ROS, the content of GSSG and the activities of XOD, MPO, and iNOS	[61]
		Coagulation and fibrinolysis	HUVECs	160 μg/mL	HUVECs pretreated with PNS were exposed to oxidized low-density lipoprotein (ox-LDL,80 mg/L) for 16 h	Inhibit effects on platelet activation and adhesion	Regulate the expression of COX-2, lead to an increase in the concentration of 6-keto-PGF1α in HUVECs, downregulate COX-1 expression and decreases the concentration of supernatant TXB2 in platelets	[76]
	Stop bleeding	Vascular homeostasis	Acellular capillaries in the retina	–	Administered orally for 1 month	Reduced the proliferation of acellular capillaries in the retina and alleviated damage to the BRB	Increase the thickness of the retinal inner nuclear layer by upregulating the expression of claudin-1 and occludin proteins	[94]
			Endothelial progenitor cell (EPC)	6.25 mg/L	EPCs were treated with different concentrations of PNS	Promoted endothelial progenitor cells angiogenesis	Increases the mRNA levels of VEGF, bFGF, VE-Cadherin, WNT3a, LRP5, β-catenin, and TCF4. After knocked down β-catenin expression	[84]
			HUVECs	200 μg/mL	HUVECs were treated with different concentrations of PNS	suppressed the effects of HG on cell dysfunction and enhanced wound healing in diabetic rats	Activate the GSK-3β/β-catenin pathway	[85]
Notoginseng R1 (NGR1)	Circulate blood	Vascular homeostasis	HF mice, H9C2 cells	7.14 mg/kg/d 40 μmol/L	In vivo, NGR1 (7.14 mg/kg/days) was orally administered for 14 days In vitro, NGR1 (40 μmol/L) was co-cultured with PA stimulation for 24 h in H9C2 cells	Improved the cardiac function and myocardial injury in mice with HF	Activate phosphorylated AMPK, promote fatty acid oxidation and inhibit lipid synthesis	[5]
			HBMEC cells	12.5–50 μM NGR1	HBMEC cells were pretreated with different concentrations of NGR1 for 12 h	Increased the migration, proliferation and tube formation of HBMECs in vitro	Activate the NAMPT-NAD-SIRT1 cascade, where SIRT1 inhibits DLL4-Notch signaling by deacetylating NICD and upregulating the expression of VEGFR-2	[29]
			HUVECs	80–110 μM	HUVECs were treated with different concentrations of PNS for 48 h	Promoted the proliferation, mobility and tube formation of HUVECs in vitro	Promote angiogenesis by activating the Ang2/Tie2 signaling pathway	[30]
			HUVECs	10, 20, 40, 80, 160 μM	HUVECs were cultured with RPMI-1640 medium containing HG for 24 h, and different concentrations of NGR1 were added for intervention	Reduced HG-induced HUVECs proliferation and viability inhibition, mitigated apoptosis, and enhanced tube formation capacity	Downregulate the MyD88/TRAF6/NF-κB pathway via upregulating miR-147a	[31]
			MCAO/R rats	20 mg kg^−1^	Administered by intraperitoneal (i.p.) injection for 7 days	Reduced the infarct size and improved neurological deficits	Ameliorate neuronal damage and inhibit glial activation	[49]
		Inflammatory response	Rat retinal capillary endothelial cells (RCECs)	10 μM	HUVECs were treated for 72 h	Reduced the production of ROS, oxidative damage and apoptosis	Regulate redox status by activating PARP	[62]
			Endothelial cells	10, 20, 40, 80, 160 μM	HUVECs were cultured with RPMI-1640 medium containing HG for 24 h, and different concentrations of NGR1 were added for intervention	Reduced HG-induced endothelial cell activity and promoted angiogenesis	Block the activation of the MyD88/TRAF6/NF-κB signaling pathway under HG conditions, thereby inhibiting oxidative stress and inflammatory responses	[31]
			Male Sprague–Dawley rats	25 mg/kg	NR1 group were administered 25 mg/kg of NGR1 monomer by gavage daily for six weeks	induced a noticeable reduction in plaque pathology	Inhibit the NLRP3/Cleaved Caspase-1/IL-1β inflammatory pathway, thereby reducing endothelial cell damage	[63]
			BALB/c nude mice (6 w), C57BL/6 mice (6 w), and Zsgreen transgenic mice	MSN-NGR1: 267 ng/kg NGR1: 40 mg/kg	Intragastric administration and intravenous injection	Improved cardiac function and angiogenesis, reduced cell apoptosis, regulated macrophage phenotype and inflammatory factors	Increase the levels of p-Akt and pERK, promoting the nuclear translocation of YAP, which targets the reduction of infarcted myocardial regions	[65]
		Coagulation and fibrinolysis	Human aortic smooth muscle (HASMCs)	0.1, 1, 10 μM	HASMCs were treated for 30 min	Decreased PAI-1 mRNA and protein expression and secretion	Suppress ERK and PKB signaling pathways	[9]
			OGD/R model to mimic HIE	10 μmol/L	Cells were administered NGR1 (10 μmol/L) when exposed to oxygen–glucose deprivation and reoxygenated	Inhibited neuron apoptosis and the expression of endoplasmic reticulum (ER) stress-associated pro-apoptotic proteins in hypoxic–ischemic encephalopathy	Inhibits the PLC/IP3R pathway and the release of Ca^2^⁺ from the endoplasmic reticulum (ER)	[78]
	Stop bleeding	Vascular homeostasis	STZ-induced diabetic rats	0.038 mg/cm^2^	All rats received topical treatment once daily for 15 consecutive days	Enhanced the healing of diabetic wounds	Enhance extracellular matrix (ECM) secretion, promote collagen growth, increasing platelet endothelial CD31 expression and decrease cleaved caspase-3 expression	[86]
Ginsenoside Rb1 (GRb1)	Circulate blood	Vascular homeostasis	HUVECs	10, 20 µM	pretrreated with GRb1 at concentrations of 10 or 20 µM for 30 min before H_2_O_2_ treatment	Reduced H₂O₂-induced endothelial dysfunction and inhibits vascular aging	Restore the decreased expression of SIRT1 and activate AMPK phosphorylation	[41]
			Female C57BL/6J mice aged 2 and 18 months	10, 20 mg/kg	GRb1 groups were administered intraperitoneal injection with GRb1 daily for 3 months	Reduced calcium deposition, collagen deposition, and the protein expression levels of collagen I and collagen III in aged mice	Mitigate age-related vascular damage by regulating the Gas6 pathway and improves chronic kidney disease-related vascular calcification via the PPAR-γ/Wnt/β-catenin axis	[51]
		Inflammatory response	Rats	40 mg/kg	Intraperitoneal injection	Alleviated pathological changes, lipid retention and thrombus formation	Reduce the adhesion and migration of monocytes by inhibiting the ROS-TNF-α-p38-VCAM-1 signaling pathway, thereby suppressing inflammation, protecting endothelial cells	[66]
			Rats	20, 40, 80 mg/kg	Intraperitoneal injection	Ameliorated myocardial I/R injury as manifested by the improvement of cardiac function indices	Downregulate the expression of Bax, Bcl-2, and cleaved-caspase 3 while activating mTOR phosphorylation, effectively inhibiting apoptosis and the inflammatory response	[67]
		Coagulation and fibrinolysis	H9C2 cells	0.5, 1.5 mmol/L	H9C2 cells were treated for 1–2 h	Reduced myocardial infarct size, mean left ventricular diastolic pressure, incidence of arrhythmia, and levels of serum creatine kinase, lactate dehydrogenase, and malondialdehyde	Attenuate MI injury in rats, partially through the downregulation of CaMKII expression	[79]
	Stop bleeding	Inflammatory response	RAW264.7 macrophages	0.5–5 mg/mL	RAW264.7 macrophages cells were treated for 24 h	Anti-inflammatory activity	Inhibit the gene expression of the pro-inflammatory mediators (IL-1β, IL-6, TNF-ɑ), chemokines (MCP-1, CXCL10), and cytokines (TGF-β1), or inhibit the expression of the proteolytic enzyme MMP-9 from maintaining strong anti-inflammatory activities	[96]
		Coagulation and fibrinolysis	HUVECs	0.1, 1, 10 μg/mL	HUVECs were treated for 24 h	Reduced oxLDL-injuring of HUVECs	Blocks the effects of ox-LDL, thereby increasing the levels of t-PA and PAI-1 and inhibiting fibrinolysis	[103]
Ginsenoside Re (GRe)	Circulate blood	Vascular homeostasis	VSMCs	0.05, 0.2, 0.8 μM	VSMCs were treated for 24 h	Antiproliferative effect on VSMCs	Activate the eNOS-NO-cGMP signaling pathway, inhibiting the proliferation of VSMCs and preventing intimal thickening and the progression of vascular lesions	[40]
		Coagulation and fibrinolysis	Human platelets	–	–	Blocked shear stress–induced platelet activation without any significant toxicity	Inhibit platelet activation through the inhibition of the vWF-GPIb/PI3K/Akt pathway	[10]
Notoginsenoside Fc (NGFc)	Circulate blood	Vascular homeostasis	Rats	3.5 mg/kg/d	Gastric gavage treatment	Delayed reendothelialization and pathological neointimal hyperplasia, and reduce autophagy in injured carotid arteries of diabetic rats	Enhance reendothelialization via autophagy activation	[87]
Ginsenoside Rg1 (GRg1)	Circulate blood	Vascular homeostasis	C57BL/6 mice	10, 20 mg/kg/d	Gavage for 4 weeks	Protect against CIH-induced vascular endothelial dysfunction	Inhibite mitochondrial ROS production	[54]
			Balloon-injured rats	4, 8, 16 mg/kg	Intraperitoneal injection with GS-Rg1 for 14 days	Inhibited vascular intimal hyperplasia	Modulate the SDF-1alpha/CXCR4, SCF/c-kit, and FKN/CX3CR1 pathways	[36]
			HUVECs	30 mmol/L	HUVECs were treated for 24 h	Reversed high-glucose induced endothelial glycocalyx disorder and increased heparanase mRNA expression in HUVECs	attenuates high glucose-induced endothelial barrier dysfunction by attenuating the associated increase in heparanase expression	[55]
		Coagulation and fibrinolysis	Mesenteric arterioles of wild type B57/b6 mice	10 mg/kg	A bolus of Rg1 was applied through the catheterized jugular vein 5 min prior to the vessel wall injury	Vessel injury-induced platelet adhesion and thrombus formation were markedly attenuated	Inhibit platelet activation by suppressing the ERK pathway, which reduces coagulation factors, obstructs thrombin production, and decreases fibrinogen deposition	[81]
Ginsenoside Rg3 (GRg3)	Circulate blood	Coagulation and fibrinolysis	Human platelet-rich plasma (PRP)	50, 100, 200, 300 μM	Platelets (108/mL) were preincubated with GRg3 in 2 mM CaCl_2_ for 3 min at 37 °C and then stimulated by thrombin (0.05 U/mL)	Reduced thrombin-stimulated platelet aggregation in a dose-dependent manner	Inhibit the influx of Ca^2+^ induced by thapsigargin and reduces the increased phosphorylation of ERK2 caused by thrombin, thereby suppressing platelet aggregation	[82]
Ginsenoside Rd (GRd)	Circulate blood	Vascular homeostasis	HUVECs	30 mM	HUVECs were treated	Ameliorated diabetes-driven vascular damage	Enhance the AMPK/SIRT1 interaction, effectively regulating oxidative stress and apoptosis	[39]
	Stop bleeding	Inflammatory response	Male Sprague–Dawley rats	–	–	Improve neurological function, reduce cerebral edema, mitigate blood–brain barrier damage, and alleviate oxidative stress and iron accumulation	Activate the cGAS/STING/DHODH signaling pathway, thereby attenuating neuronal ferroptosis both in vivo and in vitro succeeding SAH	[99]
*Panax notoginseng* triol saponins (PTS)	Circulate blood	Coagulation and fibrinolysis	Middle cerebral artery occlusion (MCAO) in rats	10 mL/kg 100, 50, 25 mg/kg	By gavage, once a day for 6 consecutive days	Reduced the size of cerebral infarction and the water content in brain tissue after ischemia–reperfusion with significantly increased number of Nissl bodies	Reduce the adhesion of platelets to damaged vascular endothelium mediated by vWF, thereby exhibiting anti-platelet aggregation and anti-thrombotic effects	[8]
Notoginseng triterpenes (NTs)	Circulate blood	Vascular homeostasis	C57BL/6 mice	40 mg/kg/day	Intraperitoneal injection for 7 days	Improved the blood perfusion and tissue morphology of random flaps to promote the survival of flaps	Activate the Beclin-1/VPS34/LC3 signaling pathway, promoting cell proliferation and migration, which effectively enhances blood perfusion and improves the survival area of flaps	[90]
Quercetin (QUE)	Circulate blood	Inflammatory response	Great saphenous vein (GSV)	200 μmol/L	GSV were treated for 24 h	Inhibited the abnormal GSV intima thickening	Reduce oxidative damage and inflammatory responses to protect the great saphenous vein (GSV) and inhibit abnormal thickening of the venous endothelium by suppressing cell proliferation	[75]
Dencichine	Stop bleeding	Vascular homeostasis	HT1080 Human fibrosarcoma cell	50 μM, 100 μM, 250 μM, 500 μM, 750 μM, 1 mM, 1.5 mM, 5 mM	HT1080 cells were treated for 24 h	Improved wound healing and induced pro-angiogenic protein expression	Affect the HIF-1α, VEGF-A and PDGF-A pathway while promoting cell proliferation, migration, invasion and MMP-2 & -9 expressions	[91, 92]
		Coagulation and fibrinolysis	Male Balb/c mice (7 or 8 weeks old and 18–22 g in weight)	0.75, 1.5, 3.0 mg/kg	Dencichine treatment groups were intraperitoneally injected for 7 days	Increased the number of circulating platelets in mice with thrombocytopenia induced by carboplatin	Regulate platelet production by promoting megakaryocyte adhesion, migration, and pro-platelet formation through the ERK1/2 and Akt signaling pathways	[108]
*Panax notoginseng* polysaccharides	Circulate blood	Inflammatory response	Human dermal fibroblast cells (HDF)	0.25–2.5 mg/mL	HDF cells were treated for 12 h	ROS and MDA contents were decreased while it aslo reversed the down-regulation of the antioxidant activity and expression of antioxidant enzyme induced by H_2_O_2_	Activate the TGF-β/Smad signaling pathway to inhibit collagen and elastin damage induced by H₂O₂, thereby protecting the skin from oxidative damage caused by hydrogen peroxide	[70]
			Myocardial cells	50, 100, 200, 400 μg/mL	Hypoxic cells were administered for 4 h	Increased the length, branching and area of mitochondria, thus effectively improving the morphological changes of myocardial cell mitochondria caused by hypoxia reoxygenation injury	Protect myocardial cells from hypoxia-induced damage by regulating mitochondrial function and can also reduce ischemia–reperfusion (IR) injury	[72]
Fermented *P. notoginseng* polysaccharides (FPNP)	Stop bleeding	Inflammatory response	HDF	0.25, 0.5, 1.00 mg/mL	HDF cells were treated for 24 h	Increased the activity of CAT, GSH-Px and SOD, and the expression of related mRNA in HDF cells	Inhibit hydrogen peroxide (H₂O₂)-induced damage to collagen and elastin by activating the TGF-β/Smad signaling pathway	[70]
Notoginsenoside Ft1 (NGFt1)	Stop bleeding	Coagulation and fibrinolysis	Human platelet-rich plasma (PRP)	5–10 μm	Platelets were preincubated with NGFt1	Enhancing aggregation induced by thrombin, collagen and ADP is peaked at 5–10 μm	Potentiate the activation of the PLCγ2-IP3/DAG-[Ca^2+^]/PKC-TXA2 signaling pathway by other stimulators, thereby contributing to the hemostatic effects of *P. notoginseng*	[104]
20(S)-panaxadiol (PD)	Stop bleeding	Coagulation and fibrinolysis	Male Wistar rats (200.0 ± 10.0 g) and Kunming mice (20.0 ± 2.0 g)	2, 4, 8 mg/kg	Subcutaneous injection of PD for 4 h	Shortenened the bleeding time of the model mouse, affected the RBC and PLT parameters of rats, reduced APTT and TT, elevated FIB concentration, while promoting platelet aggregation in human/rat-washed in vitro	Influences calcium signaling and activates the PI3K/Akt/GSK3β signaling pathway, inducing platelet aggregation and promoting hemostasis	[111]

Trauma-induced coagulopathy (TIC) is a common complication following traumatic injury, characterized by coagulation dysfunction that leads to increased bleeding tendencies or thrombus formation [113]. This condition is a major cause of mortality among trauma patients. Disseminated intravascular coagulation (DIC) on the other hand is a pathological coagulation state characterized by systemic thrombus formation and increased bleeding tendencies [114]. Although TIC and DIC have differing causes and treatment strategies, both conditions are associated with dysfunctional coagulation systems. The mechanisms behind TIC and DIC are complex and involve widespread inflammatory responses, consumption of coagulation factors, platelet dysfunction, and activation of the fibrinolytic system [115, 116]. Through a comprehensive analysis, PNS, NGR1, GRg1, NGFc, NTs, and dencichine, have been shown to contribute to effective hemostasis by targeting pathways related to vascular homeostasis. Additionally, these compounds can prevent thrombus formation caused by the release of inflammatory factors and the excessive activation of the coagulation-fibrinolytic system following severe trauma. GRd, GRb1, FPNP, NGFt1, dencichine, and PD can effectively mitigate the hypercoagulable state in the early stages of DIC through pathways related to inflammatory responses, coagulation, and fibrinolysis, thereby preventing subsequent bleeding and shock.

**Fig. 6:**
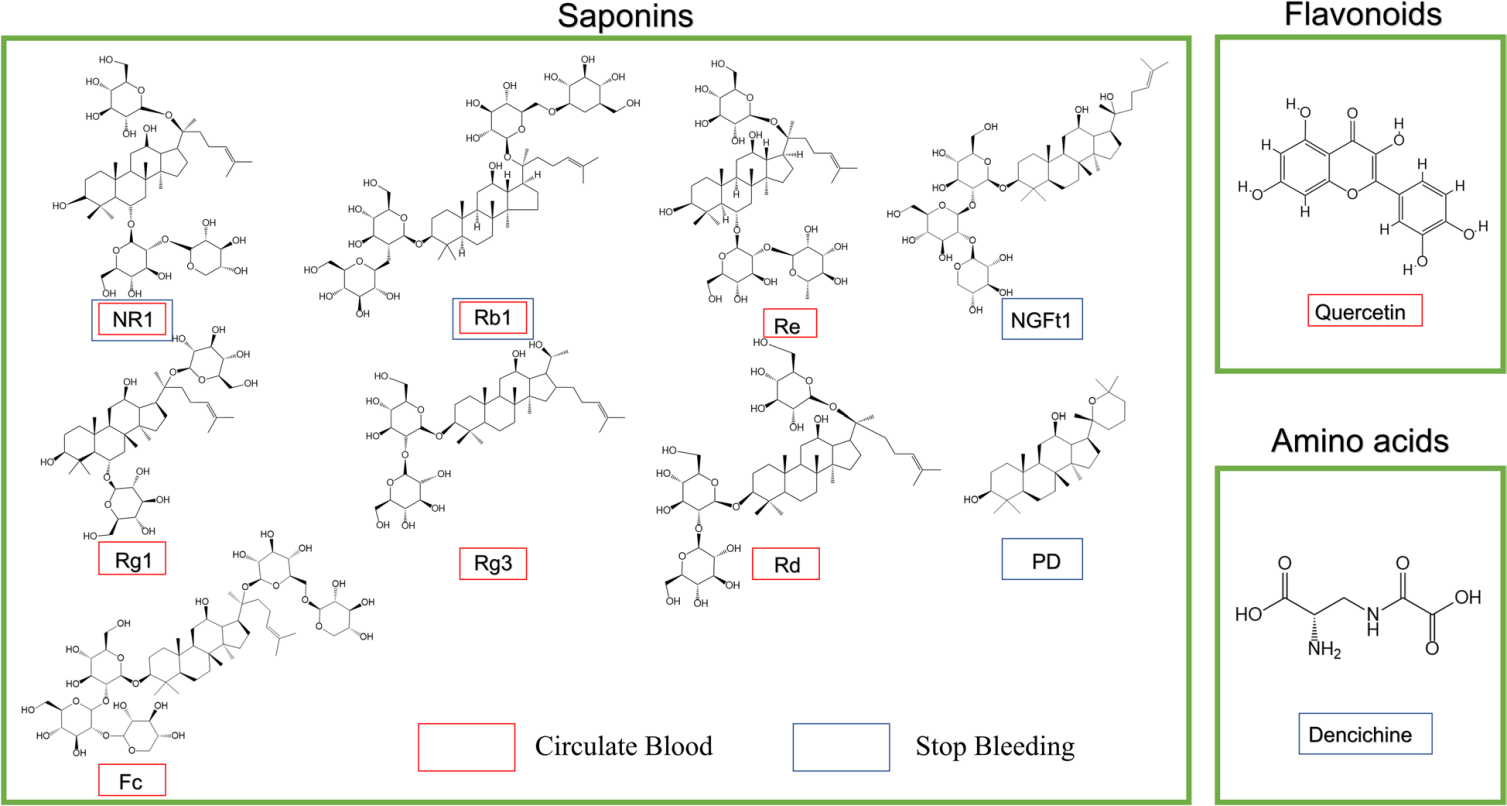
Structure of *Panax notoginseng* compounds and their effection. The structures of 11 representative compounds that can effectively promote blood circulation or stop bleeding (This figure only includes the structures of compounds with structural basis mentioned in the text)

*P. notoginseng* has long been recognized for its traditional efficacy in "promoting circulation without harming blood, achieving hemostasis without causing stasis." How can this characteristic be interpreted within the framework of modern pharmacology? As summarized earlier, the main components of *P. notoginseng* include those that promote blood circulation, those related to hemostasis, and unique components like NGR1 and GRb1, which possess both hemostatic and blood circulation-promoting properties (Fig. 6 and Table 1). Based on this, we speculate that the dual "shield and spear" effect of *Panax notoginseng* in blood regulation can be attributed to its multi-component, multi-target characteristics.

*P. notoginseng* is recognized for its ability to "promote blood circulation without causing harm," which is reflected in the following effects: Firstly, it enhances blood circulation without inducing bleeding side effects. Secondly, it exerts dual effects on both blood circulation and coagulation. Thirdly, it reduces the risk of bleeding when used in combination with anticoagulants.

Aspirin and rivaroxaban are commonly used in clinical practice as blood-thinning agents, each suited for a different type of thrombosis state [117, 118]. Although they are effective in protecting cardiovascular health, these medications also increase the risk of bleeding. In elderly patients with both cardiovascular disease and gastrointestinal bleeding, the use of antiplatelet drugs such as aspirin can elevate the risk of gastrointestinal mucosal bleeding, which in severe cases can be fatal [119]. Even low doses of aspirin can increase the risk of upper gastrointestinal bleeding by 1.37 times [120]. When combined with aspirin, total saponins from *P. notoginseng* effectively enhance the inhibition of the COX-1/TXB2 pathway and mitigate aspirin-induced gastric mucosal damage by regulating the arachidonic acid-prostaglandin metabolic pathway [121]. Additionally, it also helps control the untoward bleeding effects caused by aspirin [122]. A comprehensive analysis of the effects of *P. notoginseng* reveals that its total saponins can reduce vascular resistance and increase blood flow by relaxing vascular smooth muscle, thereby improving circulation [46]. The main components, GRe and GRb1, promote vasodilation through the COX pathway [4]. This synergy ensures that combining *P. notoginseng* with aspirin has an additive effect greater than their individual benefits in promoting circulation and reducing thrombosis. NGFt1 exerts its hemostatic effects through the Phospholipase Cγ2 signaling pathway [101]. Furthermore, *P. notoginseng* enhances hemostasis through various mechanisms, including regulating calcium influx in platelets, increasing cAMP production, and modulating TXA2 release [107]. Its bidirectional regulation of platelets is the key mechanism behind its ability to invigorate blood without causing harm in the treatment of arterial thrombosis.

Venous thrombosis primarily consists of fibrin and red blood cells. Rivaroxaban prevents the formation of fibrin by inhibiting coagulation factor Xa, effectively reducing the risk of venous thrombosis. The multi-component, multi-target nature of *P. notoginseng* further supports the safe use of rivaroxaban together with *P. notoginseng* [123]. GRg3 from *P. notoginseng* has also been shown to inhibit coagulation factor Xa [124]. Furthermore, studies indicate that TF/FVIIa, FVIII, FIX, FXI, FXII, and FXIII have all been evaluated as potential targets in animal studies or clinical trials related to the anticoagulant effects of *P. notoginseng*, with the most research focused on FIX and FXI [125]. Recent evidence suggests that FXI plays a key role in thrombosis but a secondary role in hemostasis. This finding suggests that anticoagulants that specifically target FXI may have limited effects in promoting hemostasis [126]. A study on fibrinogen suggests that mutations in the thrombin cleavage site sequence of the fibrinogen α-chain can reduce the risk of both arterial and venous thrombosis in mice. Additionally, mice expressing non-polymerizable fibrinogen maintain normal hemostatic function [127]. This finding also provides a new research direction for understanding how *P. notoginseng* promotes circulation without causing harm to the blood. Thrombin-mediated cleavage of fibrinogen to form fibrin polymers is influenced by various factors, including the regulation of calcium ion activity during thrombin formation [128], the self-assembly of fibrin monomers [129], and other related processes. Investigating whether *P. notoginseng* components affect critical steps in these processes will help elucidate its mechanism of blood circulation without causing harm in the treatment of venous thrombosis.

*P. notoginseng* is characterized by its ability to "stop bleeding without leaving blood stasis," indicating that it effectively prevents thrombosis during hemostasis. Not only does it inhibit t-PA and u-PA to reduce fibrinolysis, but its main components, NGR1 and GRg3 [9, 82], also enhance fibrinolysis through the coagulation-fibrinolytic pathway, increase plasminogen production, reduce fibrinogen deposition, and lower the risk of thrombosis. In contrast, the hemostatic drug tranexamic acid works by inhibiting fibrinolytic activity, reducing the breakdown of fibrin, and stabilizing blood clots to achieve hemostasis [130]. However, due to its anti-fibrinolytic properties, tranexamic acid carries a risk of increased thrombosis, especially when used in high doses. Deep vein thrombosis and pulmonary embolism are the most common thrombotic complications, with severe cases potentially leading to death [131]. The bidirectional regulation of the fibrinolytic pathway by *P. notoginseng* highlights it as its primary mechanism for achieving hemostasis without causing blood stasis.

Overall, the characteristic effects of *P. notoginseng* “promote blood circulation without harming it, stop bleeding without causing blood stasis"—are attributed to its multi-component, multi-target nature. When compared to current first-line clinical treatments, *P. notoginseng* appears to be relatively safe in both reducing thrombosis and promoting coagulation, offering significant potential and value for clinical application. The dual regulatory mechanism of *P. notoginseng* remains a key focus for future research.

## Conclusion

This review provides a comprehensive analysis of the active components of *P. notoginseng* and their roles in reducing thrombosis and preventing various hemorrhagic disorders. It also explores the pharmacological basis of *P. notoginseng*'s dual functions of promoting blood circulation and hemostasis, as well as the mechanisms underlying these seemingly contradictory effects. In conclusion, the key mechanism through which *P. notoginseng* promotes circulation without harming the blood lies in its bidirectional regulation of platelets during arterial thrombosis. In contrast, its effects on FXI or thrombin-mediated fibrin cleavage are well appreciated in the treatment of venous thrombosis. In addition, the primary mechanism by which *P. notoginseng* stops bleeding without forming a thrombus should focus on its bidirectional regulation of the fibrinolytic pathway.

## Data Availability

Not applicable.

## Abbreviations

*P. notoginseng*: *Panax notoginseng*
PNS: *Panax notoginseng* Saponins
NGR1: Notoginsenoside R1
GRb1: Ginsenoside Rb1
GRg1: Ginsenoside Rg1
GRe: Ginsenoside Re
GRd: Ginsenoside Rd
GRg3: Ginsenoside Rg3
NGFc: Notoginsenoside Fc
NTs: Notoginseng triterpenes
PNFS: Notoginseng flower saponins
PTS: *Panax notoginseng* Triol saponins
FPNP: Fermented *P. notoginseng* polysaccharides
NGFt1: Notoginsenoside Ft1
PD: 20(S)-panaxadiol
l-ODAP: L-β-oxalylaminoanaline
QUE: Quercetin
DVT: Deep vein thrombosis
DR: Diabetic retinopathy
FXa: Factor Xa
AS: Atherosclerosis
VSMC: Vascular smooth muscle cell
LDL: Low-density lipoprotein
RAAS: Renin–angiotensin–aldosterone system
NO: Nitric oxide
HDL: High-density lipoprotein
COX: Cyclooxygenase
VEGF: Vascular endothelial growth factor
VED: Vascular endothelial dysfunction
HG: High glucose
ERS: Endoplasmic reticulum stress
NETs: Neutrophil extracellular traps
ROS: Reactive oxygen species
TF: Tissue factor
t-PA: Tissue-type plasminogen activator
u-PA: Urokinase type plasminogen activator
BRB: Blood-retinal barrier
GCL: Ganglion cell layer
INL: Inner nuclear layer
TIC: Trauma-induced coagulopathy
DIC: Disseminated intravascular coagulation
ATP: Association of tennis professionals
cAMP: Cyclic adenosine monophosphate
ERK: Extracellular signal-regulated kinase
ECM: Extracellular matrix
NOX4: NADPH oxidase 4
PAI-1: Plasminogen activator inhibitor-1
HUVEC: Human umbilical vein endothelial cell
FXI: Factor XI
